# National survey on total-body irradiation prior to reduced-intensity stem cell transplantation in Japan: The Japanese Radiation Oncology Study Group

**DOI:** 10.1093/jrr/rrz028

**Published:** 2019-05-24

**Authors:** Hiroki Kawaguchi, Toshinori Soejima, Naoya Ishibashi, Takeshi Akiba, Masatoshi Hasegawa, Kouichi Isobe, Hitoshi Ito, Michiko Imai, Yasuo Ejima, Masaharu Hata, Keisuke Sasai, Emiko Shimoda, Masahiko Oguchi, Tetsuo Akimoto

**Affiliations:** 1 Division of Radiation Oncology, Kobe University Hospital, 7-5-2 Kusunoki-cho, Chuo-ku, Kobe City, Hyogo, Japan; 2 Department of Radiation Oncology, Kobe Proton Center, Kobe, Japan; 3 Department of Radiology, Nihon University School of Medicine, Tokyo, Japan; 4 Department of Radiation Oncology, Tokai University, Isehara, Japan; 5 Department of Radiation Oncology, Nara Medical University, Kashihara, Japan; 6 Department of Radiology, Toho University Sakura Medical Center, Chiba, Japan; 7 Department of Radiation Oncology, Kyoto Katsura Hospital, Kyoto, Japan; 8 Department of Radiology, Iwata City General Hospital, Iwata, Japan; 9 Department of Radiology, Dokkyo Medical University, Tochigi, Japan; 10 Division of Radiation Oncology, Department of Oncology, Yokohama City University Graduate School of Medicine, Yokohama, Japan; 11 Department of Radiation Oncology, Faculty of Medicine, Juntendo University, Tokyo, Japan; 12 Department of Radiation Oncology, The Cancer Institution Hospital of Japanese Foundation for Cancer Research, Tokyo, Japan; 13 Division of Radiation Oncology and Particle Therapy, National Cancer Center Hospital East, Kashiwa, Japan

**Keywords:** hematopoietic stem cell transplantation, national survey, reduced-intensity stem cell transplantation, total-body irradiation

## Abstract

Reduced-intensity stem cell transplantation (RIST) minimizes the adverse effects of traditional hematopoietic stem cell transplantation, and low-dose total-body irradiation (TBI) is administered over a short period prior to RIST (TBI–RIST). Different institutes adopt different approaches for the administration of TBI–RIST, and since no study had previously investigated this issue, a survey of the TBI schedules in Japan was conducted. In October 2015, the Japanese Radiation Oncology Study Group initiated a national survey of TBI–RIST procedures conducted between 2010 and 2014. Of 186 institutions performing TBI, 90 (48%) responded to the survey, 78 of which performed TBI–RIST. Of 2488 patients who underwent TBI for malignant disease at these institutions, 1412 (56.8%) patients were treated for leukemia, 477 (19.2%) for malignant lymphoma, 453 (18.2) for myelodysplastic syndrome, 44 (1.8%) for multiple myeloma, and 102 (4.1%) for other malignant diseases. Further, 206 (52.0%) of 396 patients (a high proportion of patients) who underwent TBI for benign disease had aplastic anemia. The TBI–RIST equipment and treatment methods were similar to those used for myeloablative regimens. Routinely shielded organs included the lungs (43.6%), eyes (50.0%) and kidneys (10.2%). The ovaries (14.1%), thyroid (6.4%) and testicles (16.7%) were also frequently shielded, possibly reflecting an emphasis on shielding reproductive organs in children. TBI–RIST was performed more frequently than myeloablative conditioning in patients with benign disease. Genital and thyroid shielding were applied more frequently in patients treated with TBI–RIST than in patients treated with myeloablative conditioning. In conclusion, this study indicates the status of TBI–RIST in Japan and can assist future efforts to standardize TBI–RIST treatment methods and to design a future multicenter collaborative research study.

## INTRODUCTION

Prior to hematopoietic stem cell transplantation, patients undergo myeloablative conditioning, which typically involves high-dose chemotherapy or total body irradiation (TBI). Conditioning regimens serve to attenuate the immunity of the recipient and facilitate the attachment of donor cells to the recipient’s bone marrow. Yet, these conditioning regimens are often contraindicated in elderly patients and patients with poor general condition due to their high therapeutic strength.

The graft-versus-leukemia (GVL) effect is a concept encompassing the way leukemia cells that persist after conditioning are attacked by donor lymphocytes that populate the recipient’s body after transplantation, leading to remission. Although reduced-intensity stem cell transplantation (RIST) is thought to induce GVL effects via the use of immunosuppressive drugs, a shortened schedule of TBI is sometimes added to the conditioning regimen in clinical practice. A retrospective analysis of the database of the Japan Society for Hematopoietic Cell Transplantation [1] indicated that adjunctive TBI–RIST did not improve outcomes. Conversely, other reports concluded that TBI–RIST led to significantly better outcomes [2, 3]. Thus far, there is no standard definition or method for TBI–RIST. Furthermore, no study to date has surveyed TBI schedules for non-myeloablative methods in Japan. Therefore, the Japanese Radiation Oncology Study Group (JROSG) designed and administered a national survey questionnaire on non-myeloablative TBI and the use of TBI in myeloablative regimens. We previously published the results of this survey regarding myeloablative TBI [4]. Here, we describe the findings for non-myeloablative TBI and compare these findings with those for myeloablative TBI presented in our previous report.

## MATERIALS AND METHODS

The survey was sent to candidate hospitals in October 2015 and reviewed patients treated between January 2010 and December 2014. The present study was conducted concurrently with an investigation of myeloablative TBI using the same dataset. We defined TBI–RIST as incorporating a total dose of <8 Gy in this survey. The questionnaire included items regarding the diseases treated with TBI and the method of TBI (including the treatment unit, treatment technique, dose and fractions, and shielded organs). All 186 Japanese institutions that performed TBI in 2015 as per the Japanese Radiation Oncology Database were approached for study participation. The survey was approved by the review board of the University Hospital Medical Information Network (UMIN000018726). The research content has been disclosed on the website of our institute and has been widely disseminated to provide opportunity to ask questions and to refuse to participate in this survey.

## RESULTS

Ninety (48%) of the 186 institutions responded to the questionnaire regarding TBI; of these, 78 indicated that they performed TBI–RIST during the study period. Table 1 indicates the distribution of patients among institutes; 80.6% of institutes performed TBI–RIST in >10 patients over the course of the 5 years.

**Table 1.** The distribution of patients among institutes

Number of treated patients	No. of institutes (%)	
1	3	(3.8)
2–5	8	(10.3)
6–10	4	(5.1)
11–30	30	(38.5)
30–100	29	(37.2)
>100	4	(5.1)
Total	78	

### Diagnosis

A total of 2884 patients received TBI–RIST at 78 institutions during the study period; of these, 2488 were treated for malignant tumors (Tables 2 and 3). Among them, 1412 (56.8%) patients were treated for leukemia, 477 (19.2%) for malignant lymphoma, 453 (18.2%) for myelodysplastic syndrome, 44 (1.8%) for multiple myeloma, and 102 (4.1%) for other malignant diseases. Among the remaining 396 patients who were treated for benign diseases, 206 were treated for severe aplastic anemia, 46 for congenital immunodeficiency, 25 for congenital metabolic abnormalities, 18 for Fanconi anemia, and 101 for other benign diseases.

**Table 2.** Summary of patients with malignant disease treated with non-myeloablative regimens (current study) and myeloablative regimens (previous study)

Diagnosis	No. of patients (%)			
	Non-myeloablative regimens		Myeloablative regimens	
Leukemia	1412	56.8	2082	(77.2)
Malignant lymphoma	477	(19.2)	378	(14.0)
Myelodysplastic syndrome	453	(18.2)	187	(6.9)
Multiple myeloma	44	(1.8)	11	(0.4)
Adult T-cell leukemia/lymphoma	23	(0.9)		
Neuroblastoma	12	(0.5)	22	(0.8)
Other	34	1.4	11	(0.4)
Unknown	33	(1.3)	7	(0.3)
Total	2488		2698	

**Table 3.** Summary of patients with non-malignant disease treated with non-myeloablative regimens (current study) and myeloablative regimens (previous study)

Diagnosis	No. of patients (%)			
	Non-myeloablative regimens		Myeloablative regimens	
Severe aplastic anemia	206	(52.0)	20	(54.1)
Congenital immune deficiency	46	(11.6)		
Inborn errors of metabolism	25	(6.3)	5	(13.5)
Fanconi anemia	18	(4.5)		
Osteomyelofibrosis	16	(4.0)	2	(5.4)
Chronic active EBV infection	14	(3.5)	7	(18.9)
Adrenoleukodystrophy	11	(2.8)		
Other	32	(8.1)	3	(8.1)
Unknown	28	(7.1)		
Total	396		37	

EBV = Epstein–Barr virus.

### Total-body irradiation schedule

With regard to the sequence of TBI–RIST, 37 institutions (47.4%) administered TBI after chemotherapy, and 19 institutions (24.3%) administered TBI before chemotherapy. Ten institutions (12.8%) selected the sequence of TBI–RIST on a case-by-case basis. There were no significant differences in the TBI schedules between myeloablative and non-myeloablative regimens.

### Treatment unit

All institutions used a linear accelerator (LINAC). The most frequent beam energy settings were 10 MV (60.2 %) and 6 MV (25.6 %). Myeloablative TBI was also highly similar among institutions, with many institutions using 10 MV (64.6%) and 6 MV (24.4%) energy settings.

### Treatment technique

Seventy institutions (89.7%) used a long source–surface distance (SSD) technique, and eight institutions (10.3%) used a moving couch technique. Among the institutions using a long SSD technique, 59 performed TBI with patients in the supine position, and 3 institutions used both supine and prone positions. Of the 8 institutions using a moving-couch technique, 4 performed TBI with patients in the supine position and 4 institutions used both supine and prone positions. Additionally, 6 of these institutions used an anterior–posterior beam arrangement. Accordingly, a majority of the 78 institutions used the supine position (63 institutions; 80.8%) and a right–left position (58 institutions; 74.4%; Table 4).

**Table 4.** Methods of total body irradiation compared with myeloablative regimens

Methods of TBI	No. of institutes (%)			
	Non-myeloablative regimens		Myeloablative regimens	
Treatment technique				
Long source–surface distance	70	(89.7)	74	(90.2)
Moving couch	8	(10.3)	8	(9.8)
Beam energy (MV)				
4	7	(9.0)	6	(7.3)
6	22	(28.2)	20	(24.4)
10	48	(61.5)	53	(64.6)
15–20	3	(3.8)	3	(3.7)
Patient position				
Supine	63	(80.8)	65	(79.3)
Supine and prone	7	(9.0)	7	(8.5)
Lateral	1	(1.3)	2	(2.4)
Others	7	(9.0)	8	(9.8)
Beam arrangement				
Right–left	58	(74.4)	60	(73.1)
Anterior–posterior	13	(16.7)	15	(18.3)
Other	7	(9.0)	7	(8.5)
Schedule of TBI (dose/fractions/days)				
7.5 Gy/3 fr/2 days	1	(1.3)		
5 Gy/2 fr/2 days	1	(1.3)		
4 Gy/1 fr/1 day	1	(1.3)		
4 Gy/2 fr/1–2 days	45	(57.7)		
3.6 Gy/2 fr/1 day	1	(1.3)		
3 Gy/1 fr/1 day	5	(6.4)		
2 Gy/1–2 fr/1 day	24	(30.8)		
Dose rate (cGy/min)				
5–9.9	28	(35.9)	33	(40.2)
10–15	34	(43.6)	42	(51.2)
>15	8	(10.3)	7	(8.5)
Unknown	8	(10.3)		
Routinely shielded organs				
Lungs	34	(43.6)	70	(85.4)
Lenses	39	(50.0)	54	(65.9)
Kidneys	8	(10.3)	1	(1.2)
Testis	13	(16.7)		
Ovary	11	(14.3)		
Thyroid	5	(6.4)		
Brain	3	(3.8)		
Cardiac	1	(1.3)		
Skin	1	(1.3)		

TBI = total body irradiation.

Regarding the number of beams in each fraction, 68 institutions (87.2%) used two beams; 4 institutions (5.1%) used four beams; 3 institutions (3.8%) used one beam; 1 institution used six beams; and 2 institutions used a variable number of beams. The treatment techniques were similar between TBI–RIST and myeloablative TBI.

### Doses and fractions of total-body irradiation

The total dose of TBI–RIST was 2–7.5 Gy, with 2 or 4 Gy being the most common doses. The number of fractions ranged from 1–3, and the total treatment duration was 1 or 2 days. The dose rate in the axis of the beam varied across institutions. Detailed results regarding the most frequently used TBI schedule at each institution are summarized in Table 4. The dose rates were similar between myeloablative and non-myeloablative TBI.

### Organ shielding

The organs that were routinely shielded during TBI are summarized in Table 4. Institutions frequently shielded the lungs (34 institutions; 43.6%) and lenses (39 institutions; 50.0%). Other common sites of shielding were the kidney (eight institutions; 10.3%), thyroid gland (five institutions; 6.4%) and heart (three institutions; 3.8%). Of note, 13 institutions (16.7%) shielded the testes and 11 institutions (14.3%) shielded the ovaries, of which 3 and 1 institutions, respectively, were treating children. Compared with myeloablative regimens, non-myeloablative regimens were less likely to shield organs, including the lungs and lenses, but the responses indicated greater attention to the shielding of reproductive organs in children.

### Duration of total-body irradiation

The duration of a single TBI session from entry to exit from the radiotherapy room was <60 min at 37 institutions (47.4%), 60 min at 33 institutions (42.3%) and >60 min at 8 institutions (10.3%). There were various approaches to TBI scheduling: 41 institutions secured irradiation time by scheduling around other patients; others set aside dedicated time for TBI in the early morning or late evening (41 institutions; 52.6%). There were no significant differences in the TBI duration or scheduling between myeloablative and non-myeloablative TBI.

## DISCUSSION

We conducted the first-ever survey on the actual status of TBI–RIST in Japan and compared our results with those of the previously reported myeloablative TBI survey. We found that, although the method was highly similar to that used in myeloablative TBI, characteristic differences were observed in the breakdown and organ shielding of cases.

Few studies have reported on the methods and state of TBI use in current clinical settings, and existing reports are characterized by significant variation between regimens. The actual conditions in Japan were investigated in three previous nationwide studies; however, the TBI methods differed across the studied institutions [5, 6]. Moreover, no independent study has compared practices between myeloablative and non-myeloablative TBI regimens. Both regimens are frequently used in tandem in clinical practice, and TBI is frequently administered as a part of conditioning regimens. Nationwide surveys of TBI–RIST methodology are lacking both in Japan and elsewhere. To our knowledge, our study is the first assessment of the current clinical position, and is important for future decisions regarding this treatment.

In recent years, recognition of the GVL effect has allowed the development of reduced-intensity conditioning regimens for hematopoietic stem cell transplantation. The GVL effect was first recognized in the 1970s [7, 8]. Although graft-versus-host disease (GVHD) generally has a negative effect on transplantation outcomes, several reports indicate that mild GVHD offers survival benefits [9, 10]. For this reason, hematopoietic stem cell transplantation is now widely applied, even among patients with complications and elderly patients aged >60 years.

Conditioning for non-myeloablative transplantation is performed using immunosuppressive drugs rather than anticancer drugs. Early studies demonstrated the efficacy of purine analogues as conditioning agents [11], and the development of combination therapy with fludarabine and an alkylating agent is currently underway. In Japan, the Hematopoietic Stem Cell Transplantation Society guidelines for non-myeloablative transplantation treatment include representative conditioning regimens. Regarding the use of TBI, a low-dose regimen of <4 Gy is recommended regardless of the underlying disease, disease stage, stem cell source, and the presence of any comorbidities at the time of transplantation. At present, no prospective studies have examined or established the efficacy of non-myeloablative conditioning regimens; however, in the Japanese clinical sphere, a report by Nakasone *et al.* indicated positive outcomes related to engraftment dysfunction in cases wherein non-myeloablative conditioning was performed first, and in three-quarters of cases that underwent TBI before and after RIST [12]. Accordingly, there is a tendency to perform TBI for the merits associated with engraftment dysfunction. In addition, the TBI in this report has been categorized into low-dose TBI and high-dose TBI, based on a cut-off dose of 8 Gy, and low TBI accounted for approximately one-third of the TBI. The period covered in this report was 2006 to 2013. In this survey targeting cases from 2010 to 2014, TBI–RIST was performed in approximately the same number of cases as was myeloablative TBI [4]. Considering the results, although a simple comparison cannot be conducted, the prevalence of TBI–RIST may be increasing. However, the timing of TBI is determined by each institution: in cases of its necessity, timing and dose fractionation are aspects that are strongly influenced by the medical opinion of the hematologist, regardless of whether TBI is used for myeloablative or non-myeloablative transplantation, even now.

We previously published a separate report regarding TBI regimens and methods for myeloablative pretreatment based on the survey used in this study. In general, we found that the equipment and treatment methods were generally similar between TBI–RIST and myeloablative regimens; therefore, we have not included a discussion comparing these items in the present report. Items specific to TBI–RIST are individually discussed below.

First, TBI–RIST was used to treat 396 benign cases (13.7% of all TBI–RIST cases), while benign cases accounted for only 1.4% of myeloablative procedures. Aplastic anemia accounted for similar percentages (about 50%) of benign cases treated with myeloablative and non-myeloablative regimens. Conventional monotherapy cyclosporine is used as a conditioning agent for aplastic anemia, and in combination with 2 Gy TBI decreased the incidence of engraftment failure in homogeneous hematopoietic stem cell transplantation from a human leukocyte antigen-matched unrelated donor [13]. There are no definitive findings regarding the optimal dose of TBI for aplastic anemia, with reported doses ranging from 2 to 10 Gy in the Japanese literature [14].

Myelodysplastic syndrome is another common complication of conditioning; however, it is thought that the reported incidence of this complication is influenced by the exclusion of these cases from conditioning for myeloablative transplantation, particularly in patients with high disease susceptibility related to age. One report combined RIST with low-dose TBI (2 Gy) in patients who were ineligible for conventional hematopoietic stem cell transplantation [15].

Next, several institutions performed dose fractionation of either 4 Gy or 2 Gy within 2 days. The Japan Society for Hematopoietic Cell Transplantation Guidelines recommend combined regimens with low-dose (2 Gy) TBI, and various reports have described these regimens [15, 16] and combination regimens with 4 Gy TBI, including in Japan [17–19].

Finally, as far as we were able to determine in our literature review, there were no reports on the necessity of organ shielding in TBI–RIST or comparisons of adverse events based on the presence or absence of organ shielding. Yet, approximately half of the institutions that administered conditioning regimens for the lungs and eyes also performed organ shielding. Additionally, while the current study was unable to provide full clarification based on respondents’ answers, it was inferred that, for institutions that reported the use of organ shielding, it was mainly used in children, particularly for the protection of reproductive organs. Nonetheless, the current findings do not provide sufficient evidence to enable a discussion of the advantages or disadvantages of organ shielding. Future studies on this topic are necessary.

The present study had some limitations. The first relates to the purpose of the survey, which was not to summarize the most effective regimen, survival rate, and treatment outcomes, but rather to clarify the current state of TBI use in Japan. As such, it is noteworthy that the use of a particular method by a large proportion of institutions is not indicative of efficacy. Second, we cannot exclude the possibility of sampling bias, because responses were obtained from only half of the institutions reporting the use of TBI. Future research in the form of a multi-institutional collaborative effort is needed to inform the use and efficacy of TBI in hematopoietic stem cell transplantation. A recent report from a Japanese institution suggested that TBI in intensity-modulated radiation therapy using helical tomotherapy was useful for shielding organs at risk [20]. In addition, some institutions may use methods other than LINAC to perform TBI; hence, it is necessary to regularly investigate the actual state of TBI in the future, with the cooperation of as many institutions as possible.

## CONCLUSION

In conclusion, this survey was able to clarify the actual status of TBI–RIST in Japan. It is of importance that this is the first study of its kind; it is likely that the issues exposed will be useful in the design of future prospective studies on TBI–RIST combination regimens.
